# Spatial and Temporal Patterns of *Campylobacter* Infection and Projected Habitat Suitability of Dominant *Campylobacter* Species in Eastern Ethiopia

**DOI:** 10.1029/2024GH001146

**Published:** 2026-04-10

**Authors:** Xiaolong Li, Amanda E. Ojeda, Loic Deblais, Bahar Mummed Hassen, Mussie Bhrane, Gireesh Rajashekara, Song Liang, Jemal Yousuf Hassen, Sarah L. McKune, Arie H. Havelaar, Jason K. Blackburn, CAGED Research Team

**Affiliations:** ^1^ Department of Environmental and Global Health College of Public Health and Health Professions University of Florida Gainesville FL USA; ^2^ Emerging Pathogens Institute University of Florida Gainesville FL USA; ^3^ Department of Microbiology and Cell Science Institute of Food and Agricultural Sciences University of Florida Gainesville FL USA; ^4^ Center for Food Animal Health, Department of Animal Sciences, College of Food, Agricultural, and Environmental Sciences The Ohio State University Wooster OH USA; ^5^ Haramaya University Haramaya Ethiopia; ^6^ Center for African Studies University of Florida Gainesville FL USA; ^7^ Global Food Systems Institute University of Florida Gainesville FL USA; ^8^ Spatial Epidemiology and Ecology Research Laboratory, Department of Geography University of Florida Gainesville FL USA

## Abstract

*Campylobacter* is the most common bacterial cause of foodborne illness globally. Both symptomatic and asymptomatic infections with *Campylobacter* species have been associated with growth faltering of children in low‐resource settings, while previous prevalence studies primarily focused on diarrheal disease in children. Here, we leverage the data collected from the *Campylobacter* Genomics and Environmental Enteric Dysfunction (CAGED) project to characterize the spatial patterns of *Campylobacter* infections among infants with or without diarrhea in rural Eastern Ethiopia. Randomly enrolled infants (*n* = 106) were followed from birth to around 13 months, with fecal samples collected monthly. Livestock feces, drinking water, and soil samples were collected biannually. *Campylobacter* was detected and quantified using genus‐specific PCR and species‐specific PCR for four species. We employed a spatial filtering approach using genus‐specific data to generate smoothed prevalence surfaces by month and age group. Temporally, an upward trend of prevalence was observed as the children grew older. Spatially, high‐prevalence areas were distributed across the whole study area. To relate disease risk to environmental conditions, we used ecological niche modeling with MaxEnt to estimate habitat suitability of the genus *Campylobacter* and two dominant species identified by PCR results. Elevation, vegetation index, and slope were the most important contributors, and all distribution models suggested areas in the north were more likely to support the pathogen. These results inform *Campylobacter* infection patterns and identify target areas with higher risk of *Campylobacter* in low‐resource settings. This further contributes to developing effective intervention strategies in the future.

## Plain Language Summary

Campylobacters are species of bacteria that cause food‐borne illnesses, leading to clinical signs including diarrhea, fever, and abdominal pain. These infections are particularly concerning in low‐resource settings where they can significantly affect the health and long‐term development of young children. Understanding how these infections spread and identifying high‐risk areas can help in developing effective prevention strategies. Here, we investigated the prevalence of Campylobacter infections in infants in rural Eastern Ethiopia, both with and without diarrhea, to understand their spread and impact on child growth. We followed 106 infants from birth to about 13 months, collecting fecal samples monthly and environmental samples from livestock, water, and soil twice a year. Campylobacter was detected using polymerase chain reaction (PCR) genetic tests, and the infection rates were mapped over time and across different locations. We found that as children aged, the prevalence of Campylobacter increased, and certain areas consistently showed higher infection rates. Using environmental data, we also predicted which areas were most suitable for the bacteria, identifying elevation, vegetation, and slope as key factors. These findings highlight regions with higher infection risks, offering valuable insights for future public health interventions to prevent and control Campylobacter infections in vulnerable populations.

## Key Points

- Prevalence of *Campylobacter* infections among infants in rural eastern Ethiopia showed an upward trend in their first year of life
- *Campylobacter* infections were distributed across the study area, with some regions having persistently higher prevalence over time
- MaxEnt‐based distribution models predicted northern areas were more likely to support the *pathogen at both the genus and species level*

## 1 Introduction

Species in the genus *Campylobacter* are known as the most common bacterial cause of foodborne illness worldwide and contribute to approximately 166 million cases each year in both industrialized and developing nations (A. H. Havelaar et al., 2015). Furthermore, a body of evidence shows that *Campylobacter* infections have been associated with stunting and environmental enteric dysfunction (EED), a subclinical disorder characterized by inflammation and altered function in the small intestine (Keusch et al., 2014), among children under 5 (CU5) in low‐resource settings (Amour et al., 2016; Haque et al., 2019; Lee et al., 2013; Rogawski et al., 2018). Both symptomatic and asymptomatic infections with *Campylobacter* spp. are linked to these health effects.

Diarrhea that can be attributed to *Campylobacter* colonization is often observed in Africa, and previous studies reported the prevalence of *Campylobacter* infection among CU5 ranged from 1.7% to 15.5% in several African countries (Gahamanyi et al., 2020). As most of the samples collected in these studies came from children with diarrhea, the actual prevalence of *Campylobacter* colonization among CU5 is likely to be underestimated in Africa, given that asymptomatic *Campylobacter* infections are often very common in these settings (Amour et al., 2016; Platts‐Mills & Kosek, 2014).

Over 25 species of *Campylobacter* have been identified, of which *Campylobacter jejuni* and *Campylobacter coli* are the two most commonly reported species associated with human illnesses (Kaakoush et al., 2015). Due to the clinical importance of these two species, traditional culture methods were designed to selectively isolate these two species, resulting in a large body of knowledge on disease burden and clinical manifestations specific to *C. jejuni* and *C. coli* (François et al., 2018). Owing to the development of molecular techniques and innovative isolation approaches, a number of non‐*C. jejuni/coli* species (i.e., *Campylobacter* species other than *C. jejuni* and *C. coli*) have drawn increasing attention and have been recognized as “emerging *Campylobacter* species” (Man, 2011). They include *C. concisus*, *C. lari*, *C. upsaliensis*, and *C. ureolyticus*. The Etiology, Risk Factors and Interactions of Enteric Infections and Malnutrition and the Consequences for Child Health and Development (MAL‐ED) project used both enzyme immunoassay (EIA) and polymerase chain reaction (PCR) to detect *Campylobacter* in stool samples and found that EIA identified a broader range of *Campylobacter* spp. (Platts‐Mills et al., 2014). In a subset of *Campylobacter*‐positive samples from MAL‐ED, non‐*C. jejuni/coli* species accounted for around 30% of detections by EIA. Moreover, association analysis of *Campylobacter* infection on child growth revealed that infection with *Campylobacter* species detected by EIA showed a stronger association with child stunting compared to *C. jejuni/coli* infection detected by PCR specific for the two species (Haque et al., 2019), implying the underlying role of non‐*C. jejuni/coli* species in affecting poor child growth. Our previous cross‐sectional study on *Campylobacter* colonization and child health outcomes (i.e., EED and stunting) among children in eastern Ethiopia suggested that non‐*C. jejuni/coli* species including *C*. *hyointestinalis, C*. *fetus*, and *C*. *concisus* detected by meta‐total RNA sequencing (MeTRS) were more prevalent in child stool samples than *C. jejuni/coli* (Chen, McKune, et al., 2021; Terefe et al., 2020).

Recent studies also showed the emergence of a new species, “*Candidatus Campylobacter infans*” (*C. infans*) isolated from stool samples of children under 2 years in low‐resource settings along with other non‐*C. jejuni/coli* species (Bian et al., 2020; Garcia Bardales et al., 2022; Parker et al., 2022). All this evidence draws our focus in this study to the broader species within the genus *Campylobacter* rather than just *C. jejuni/coli*.

Given the high disease burden, a great effort has been made to identify the risk factors and transmission pathways of *Campylobacter* bacteria (specifically *C*. *jejuni* and *C*. *coli*), as well as their associations with child health outcomes in Africa (Amour et al., 2016; Budge et al., 2020; El‐Tras et al., 2015; Lee et al., 2013; Lengerh et al., 2013; Luby et al., 2018; McQuade et al., 2020; Null et al., 2018; Schiaffino et al., 2021; Stewart et al., 2018). However, these patterns have yet to be fully addressed spatially or temporally. For instance, did areas with a high prevalence of *Campylobacter* infection among children persist in space and time? Did the geographic focus of *Campylobacter* infections shift over time within a specific pediatric population and how? The dynamics of such infection patterns over space and time in African countries are seldom discussed in the literature. Addressing these questions will greatly facilitate the development of effective and sustainable intervention strategies to combat this enteric pathogen among young children in low‐ and middle‐income countries (LMICs).

The spatial/temporal pattern analysis of *Campylobacter* infections will help capture the heterogeneity of infections on the landscape. Still, we also need to understand which environmental conditions are suitable for the pathogen to infect young children. One approach to modeling such suitability is ecological niche modeling. MaxEnt, which employs a maximum entropy approach, is one of the most commonly used methods for ecological niche modeling (Elith et al., 2006; Elith & Leathwick, 2009). Generally, ecological niche models estimate the ecological niches of species in variable space and project the potential geographic distribution across the landscape (Alexander et al., 2012; Blackburn, 2010). MaxEnt uses presence‐only data and applies a machine learning algorithm to estimate a target probability distribution that has a maximum similarity to the prior distribution (represented by random background samples) while taking account of the constraints imposed by the incomplete information derived from the presence locations (Elith et al., 2011; Merow et al., 2013; S. J. Phillips et al., 2006). MaxEnt has been widely applied in diverse ecology, evolution, and conservation biology scenarios to study species' distributions, richness and biodiversity (Elith et al., 2006). Similar to other types of species, a pathogen also has its corresponding ecological niche characterized by ecological parameters (Mullins et al., 2011). Within such a conceptualized N‐dimensional space, the pathogen can be maintained. Previous studies regarding relationships of environmental variables and *Campylobacter* species primarily focused on the potential impact of the natural environment on human campylobacteriosis cases (Arsenault et al., 2012; Kuhn et al., 2020; Louis et al., 2005; Soneja et al., 2016; Weisent et al., 2014), but did not investigate the species niches for *Campylobacter* in certain settings. Here, we used MaxEnt to estimate the habitat suitability of *Campylobacter* species in a low‐resource setting of Ethiopia.

The *Campylobacter* Genomics and Environmental Enteric Dysfunction (CAGED) project is a longitudinal study conducted in rural Eastern Ethiopia to examine the reservoirs of *Campylobacter* spp. infections among infants and their interactions with child health outcomes (A. H. Havelaar et al., 2022). In the present work, by using the data collected for the CAGED project, we aim to:

1. Characterize the spatial and temporal patterns of *Campylobacter* infection among children under 1 year in Eastern Ethiopia, and
2. Estimate the habitat suitability of dominant *Campylobacter* species in the study area using ecological niche models.

## 2 Materials and Methods

### 2.1 Study Design and Protocols

**Figure 1:**
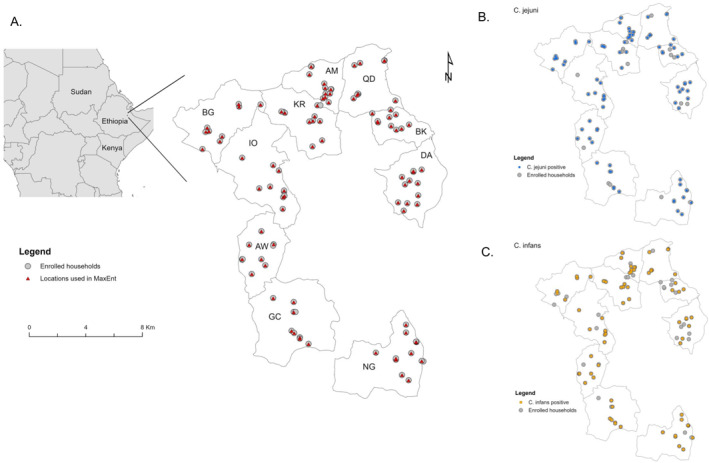
Study area and enrolled households in the *Campylobacter* Genomics and Environmental Enteric Dysfunction (CAGED) project. Gray dots represent the locations of 106 enrolled households. Panel (a) black triangles represent the presence locations used to build MaxEnt models at the genus level (see Section 2). Panels (b, c) blue and red dots represent the presence locations for two dominant *Campylobacter* species, *C. jejuni* and *C. infans*, respectively. Abbreviations: AW—Adele Walta; AM—Amuma; BK—Bachake; BG—Biftu Geda; DA—Damota; GC—Gobe Challa; IO—Ifa Oromia; KR—Kuro; NG—Nageya; QD—Qerensa Dereba.

The detailed CAGED study design and protocols have been described elsewhere (A. H. Havelaar et al., 2022). Briefly, this longitudinal study was conducted in the rural Haramaya woreda (district equivalent) of Eastern Ethiopia where 10 kebeles (the smallest sub‐district administrative unit in Ethiopia) were selected as the source population (Figure 1). A total of 106 infants were randomly enrolled from the source population at birth and followed up until approximately 13 months of age. Enrollment started in December 2020 and was completed in May 2021. Up to 20 newborns were enrolled per month until the desired sample size was achieved. Starting the second month of the longitudinal study, both enrollment and follow‐ups took place simultaneously, resulting in temporally disjoint follow‐up periods for children enrolled at different months. Fecal samples were collected from enrolled infants monthly and from their mothers and siblings as well as, livestock (i.e., chicken, cattle, goat, and sheep) present in or around the homestead, and the environment (soil and drinking water) biannually. Sample collection was conducted from December 2020 to June 2022, and a total of 1073 child fecal samples and 1361 environmental samples were collected.

In the laboratory, DNA extraction and quantification were performed, followed by genus‐specific Taqman real‐time PCR targeting 16S rRNA to detect and quantify *Campylobacter* (*n* = 2,717 samples). A positive *Campylobacter* infection was defined as the cycle threshold (Ct) value of a child fecal sample being less than 35 (Deblais et al., 2023). To assess the diversity of *Campylobacter* infections detected in our study population, species‐specific SYBR Green real‐time PCR was performed. Species‐specific primers targeting *hipO* and *cpn60* were used. A total of four *Campylobacte*r species including thermophilic and nonthermophilic species were tested, including: Candidatus *C. infans, C. jejuni. C. upsaliensis* and *C. lari*. Other species were rarely, if ever, detected in infant stools. A cutoff Ct value of 35 was used to detect *Campylobacter species* in the human stool, animal feces, and soil (*n* = 2,045 samples). Nuclease‐free water was utilized as a negative control for all qPCR testing. Further details on laboratory methods are provided in Deblais et al. (2023) and in a separate manuscript describing the species composition of *Campylobacter* (Ojeda et al., 2025).

### 2.2 *K*‐Means Clustering for Dividing Age Groups

As previous evidence showed that the prevalence of *Campylobacter* infections differed between age groups of children under 2 (Amour et al., 2016), we divided our study population into four age groups to account for the confounding role of child age in *Campylobacter* infections and to investigate the infection patterns among different age groups.

*K*‐means clustering is a partitioning method that divides a data set into *k* clusters through an iterative process of searching for the centroid (mean) in each cluster that minimizes the within‐cluster sum of squares (Hartigan & Wong, 1979). Here, we first calculated the age (days) of the children when their fecal samples were collected in each home visit and then partitioned the child age data set into four groups. We opted for four clusters to align with a previous study on the same population, which divided children into quartiles based on age (Chen et al., 2024). While the previous study used quartiles as a straightforward statistical method, we sought to adopt a more data‐driven approach that accounts for the natural structure of our data set. The use of four groups allows for direct comparability with the prior study while providing a clustering solution reflective of the data's inherent patterns. *K*‐means clustering was performed in R software using the *kmeans* function.

### 2.3 Spatial Filtering Approach

To estimate the infection pattern of *Campylobacter* among infants over the study area and during the study period, a spatial filtering approach in the Disease Mapping and Analysis Program (DMAP, available for download at http://www.uiowa.edu/~gishlth/DMAP/) was employed to generate smoothed prevalence surfaces of *Campylobacter* infections based on the monthly genus‐specific qPCR data defining infection status.

DMAP is an exploratory spatial analysis tool first applied to estimate local birth defect rates with georeferenced birth data in Iowa (Rushton & Lolonis, 1996). A detailed description of the spatial filtering in DMAP can be found elsewhere (Lentz et al., 2011). Briefly, DMAP applies a circular spatial filter with a radius defined by the user, traversing grid points situated over the landscape. The prevalence at each grid intersection point is then calculated based on the number of presence/absence points falling within the filter circle which can cover multiple grid cells. In this analysis, we generated a gridded surface covering the study area with 250‐m grid cells. To determine the radius of the spatial filter, we first calculated the average k‐nearest neighbor distances at different order (*k* = 1, 2, …, 10) using the geographic coordinates of the 106 enrolled households in R. While exploring larger orders (*k* > 2), the DMAP software encountered limitations in handling these distances effectively on our relatively small landscape, resulting in computational issues and unreliable outputs. To address this, we selected *k* = 2 (780.53 m) as it represented a practical trade‐off, providing sufficient spatial resolution while ensuring compatibility with the software and reliability of the analysis. This approach allowed us to capture meaningful spatial patterns within the constraints of our data set and analysis tools.

The presence/absence of *Campylobacter* infection data were organized by age group, and DMAP estimation was performed for each month in each age group. To ensure that enough points could be used in each DMAP run, we removed those months with less than 15 data points (presence and absence records). Grid point prevalence estimates for each month and age group were then interpolated using inverse distance weighting (IDW) with a power of 2 in ArcMap 10.8.2 (ESRI; Redlands, CA) to generate continuous surfaces (raster files) of prevalence rates. To identify areas of increased prevalence, IDW‐interpolated rates were mapped as standard deviations (SD) from the mean.

Owing to the limited number of data points available for the two dominant species in comparison to the genus level, similar analyses focusing on these two specific species were not conducted.

### 2.4 Space Time Analysis of Moving Polygons (STAMP)

**Figure 2:**
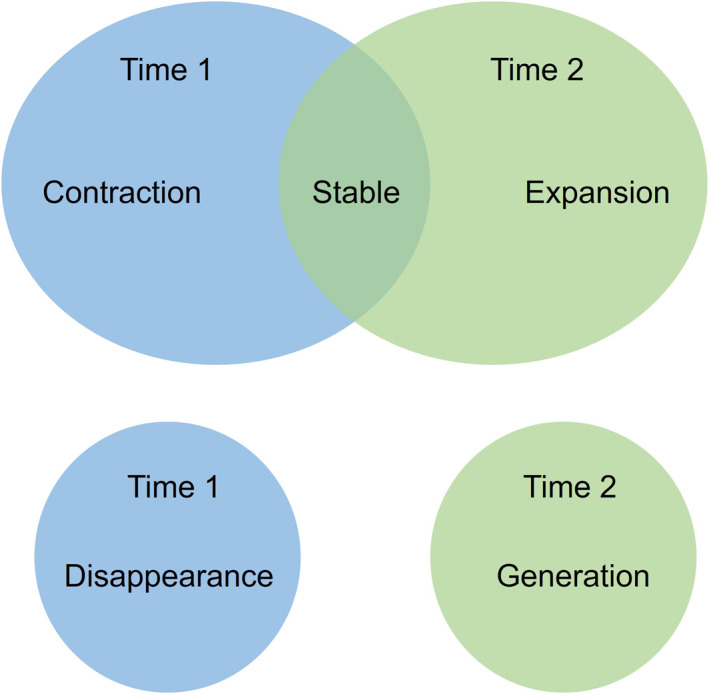
Types of polygon movement events defined in the Space‐Time Analysis of Moving Polygons (STAMP). Blue circles and green circles represent areas at Timepoint 1 and Timepoint 2, respectively. The overlapping region of blue and green circles is defined as stable, which exists at both time points, while the rest parts adjacent to stable are contraction type (only present at Timepoint 1) and expansion type (only present at Timepoint 2). Disappearance and generation are events independent from other areas at either timepoint.

Areas of prevalence greater than 1.5 SD were used to identify persistent hot spots of *Campylobacter* infections over time for each age group through the STAMP approach. STAMP was designed to delineate the space‐time change of polygon movement events between two consecutive time points T1 and T2 (Robertson et al., 2007). The types of polygon movement events can be categorized as stable, extraction, expansion, generation, and disappearance based on the intersection of polygons at T1 and T2. Stable type refers to polygons that are present at both time points. In this study, a stable polygon is an area where the prevalence of *Campylobacter* infection has been persistently high for two consecutive months. If polygons only exist at either time point, they are categorized as generation (only present at T2) or disappearance (only present at T1). That is, the area of high prevalence only exists in one of the two consecutive months. Extraction and expansion are two special cases of generation and disappearance; extraction is the disappearance events that are adjacent to stable events, while expansion is the generation events adjacent to stable events (Figure 2).

Smoothed prevalence surfaces from DMAP were first reclassified by SD and converted to polygons in ArcMap, and the STAMP analysis was then performed for each age group separately using R package *stampr* (Long et al., 2018). Stable areas of high prevalence of *Campylobacter* infection were the focus of this study and were defined as any polygons that persist for more than two consecutive months. Since STAMP only deals with two consecutive months for each run, stable polygon layers obtained from multiple runs were overlaid by age group, and intersections of the stable layers were calculated using the “Intersect” tool in the toolbox of ArcMap.

### 2.5 Environmental Variables for Ecological Niche Modeling

The DMAP analysis was used to map the heterogeneity of infection on the landscape but cannot be used to relate disease risk to environmental conditions. For this purpose, we used ecological niche modeling with MaxEnt, including 13 covariates covering topography, climate, vegetation, soil, and human population density. These were selected based on relevant literature modeling the prevalence of diarrhea‐related infections locally and globally (Colston et al., 2022; Reiner et al., 2020; Sanderson et al., 2018) (Table 1). Raster layers of elevation, slope, and population counts (2020) for Ethiopia were downloaded from WorldPop (https://www.worldpop.org/) with a spatial resolution of 100 m. Daily land surface temperature (LST) and 16‐day normalized difference vegetation index (NDVI) grids were obtained from the Terra Moderate Resolution Imaging Spectroradiometer (MODIS) Land Surface Temperature/Emissivity Daily (MOD11A1) Version 6.1 product (https://lpdaac.usgs.gov/products/mod11a1v061/) and MODIS Vegetation Indices (MOD13Q1) Version 6.1 product (https://lpdaac.usgs.gov/products/mod13q1v061/), respectively. The minimum, maximum, and mean of these two products over the study area in 2021 were calculated using the Code Editor in Earth Engine (https://code.earthengine.google.com/), and 6 covariates for daily LST and 16‐day NDVI were generated correspondingly. Grids representing soil features, namely the proportion of clay particles in the fine earth fraction, organic carbon content in the fine earth fraction, and soil pH, were downloaded from SoilGrids version 2.0 (https://soilgrids.org/) at a 250‐m spatial resolution. Wind speed at 100 m above ground data were from the Global Wind Atlas (https://globalwindatlas.info/en/). As these environmental layers had varying spatial resolutions, all were resampled to 250 × 250 m grids and cropped to the boundary of the study area using the “raster” package in R.

**Table 1:** Environmental Covariates Used in the MaxEnt Models

Variable	Unit	Spatial resolution	Data source
Elevation	m	100 m	WorldPop (www.worldpop.org/)
Maximum daily land surface temperature, 2021	°C	1 km	USGS MODIS product (MOD11A1)
Mean daily land surface temperature, 2021	°C	1 km	USGS MODIS product (MOD11A1)
Minimum daily land surface temperature, 2021	°C	1 km	USGS MODIS product (MOD11A1)
Maximum 16‐day NDVI, 2021	N/A	250 m	USGS MODIS product (MOD13Q1)
Mean 16‐day NDVI, 2021	N/A	250 m	USGS MODIS product (MOD13Q1)
Minimum 16‐day NDVI, 2021	N/A	250 m	USGS MODIS product (MOD13Q1)
Population count, 2020	person	100 m	WorldPop (www.worldpop.org/)
Slope	degree	100 m	WorldPop (www.worldpop.org/)
Proportion of clay particles in the fine earth fraction	g/kg	250 m	SoilGrids 2.0 (https://soilgrids.org/)
Soil organic carbon content in the fine earth fraction	dg/kg	250 m	SoilGrids 2.0 (https://soilgrids.org/)
Soil pH	pH × 10	250 m	SoilGrids 2.0 (https://soilgrids.org/)
Wind speed at 100 m above ground	m/s	250 m	Global Wind Atlas (https://globalwindatlas.info/en/)

### 2.6 Ecological Niche Modeling

MaxEnt (Version 3.4.4) software (Philips et al., n.d.) was used to model the potential geographic distribution of the genus *Campylobacter* as well as two dominant species identified by PCR results, *C. jejuni* and *C. infans*, over the study area (Deblais et al., 2023). To better understand the pathogen's ecological context and to enhance the predictive accuracy of ecological niche models, we used the environment samples collected from drinking water, soil, and livestock feces in enrolled households for modeling. The purpose of these samples in this study was not to characterize the diversity or distribution of *Campylobacter* species, but to determine the presence or absence of *Campylobacter* at each household. If *Campylobacter* was detected in any one of the environmental samples collected from a given household, that household was assigned a positive status for the purposes of ecological niche modeling. Details on the composition and distribution of *Campylobacter* species in these environmental samples were presented in a separate study from our research team (Ojeda et al., 2025).

As *Campylobacter* was identified by genus‐specific qPCR at least once from the environmental samples collected at each household (Deblais et al., 2023) during the study period, we initially had 106 presence sites available for the ecological niche modeling at the genus level. In a presence/absence framework, data points need to be spatially filtered to one point per grid cell as the resolution of the climatic conditions (here 250 m) (Joyner et al., 2010). This filtering reduced our data set to 93 presence locations (Figure 1a). Similarly, a total of 78 and 69 presence locations were obtained for *C. jejuni* and *C. infans*, respectively (Figures 1b and 1c). For all MaxEnt models, the maximum number of background points was set as 1000 due to the relatively small area of the landscape and the number of grid cells. The input data set was randomly split into 80% for training and 20% for testing, and we ran 10 replicates with a bootstrapping procedure to account for the variations between models using different sets of training/test points.

We retained all environmental covariates in the final model for two reasons. First, their inclusion ensured consistency with previous studies, facilitating comparability with existing research. Second, this approach allowed for the exploration of potential differences in covariate contributions between genus‐level and species‐level models, where some covariates might have varying impacts. By including all covariates, we maintained consistency across comparative analyses. Model performance was evaluated by the area under the receiver operating curve (AUC). An AUC of 0.5 indicates the model performance is no better than random, and, empirically, values greater than 0.8 are used as an indicator of good prediction (Kumar et al., 2020).

The average of these 10 bootstrapped models was used to estimate the habitat suitability of *Campylobacter* genus and each of the two species on the landscape. To make the interpretation of the suitability map easier, we described the output as “low” suitability at 0.25, “medium” suitability at 0.5, and “high” suitability at 0.75 following similar studies (Bartlow et al., 2022; Gorris et al., 2021). The uncertainty of our models was presented by the standard deviation of habitat suitability at each pixel for the 10 replicates.

We used the mean values of the built‐in permutation importance and percent variable contribution output from the 10 bootstrapped models as the metric to identify the contribution/importance of environmental covariates. In each iteration of the model training process, MaxEnt monitors the change of gain, a metric of goodness‐of‐fit comparing the average log probability of the presence samples against a uniform distribution where the gain is defined as 0, after modifying the coefficient of a specific covariate. The greater the gain is, the higher the average likelihood of the presence samples in the model. The percent contribution is obtained by converting the total gain assigned to each environmental covariate into a percentage at the end of the training process (S. J. Phillips, 2021). Unlike percent contribution, permutation importance of each covariate only depends on the final MaxEnt model. Values of a specific environmental variable are randomly permuted among both presence and background training points, and the resulting decrease in training AUC is used to measure the extent to which the model depends on that variable. A large decrease means that the corresponding variable is important to the final model. Values of decrease for all environmental variables are converted to percentages after normalization as their permutation importance.

## 3 Results

### 3.1 Prevalence of *Campylobacter* Infections by Age Group

**Figure 3:**
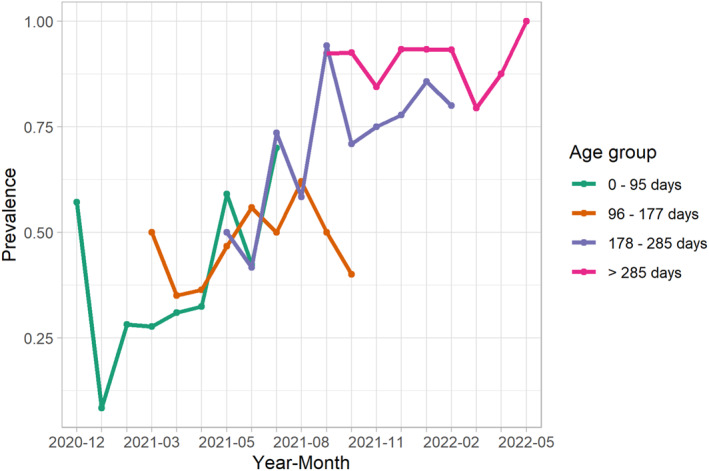
Prevalence of *Campylobacter* infections among infants in 10 kebeles of Ethiopia from December 2020 to June 2022 by age group. Note, age group does not directly correspond to month due to the enrollment schedule; no more than 20 children were enrolled per month.

The minimum and maximum child age when fecal samples were collected were 7 and 376 days, respectively. The *k*‐means clustering gave us four age groups: 0–95 days, 96–177 days, 178–285 days, and >285 days. The monthly prevalence of *Campylobacter* infections in the first age group (0–95 days) ranged from 0.08 to 0.70, covering the months of December 2020 to August 2021 (Figure 3). The monthly prevalence of *Campylobacter* infections in age Group 2 (96–177 days) ranged from 0.35 to 0.62 from March 2021 to November 2021. For older age groups, the monthly prevalence increased further: 0.42–0.94 for age Group 3 (178–285 days) and 0.79–1.0 for age Group 4 (>285 days). No apparent seasonality was observed in each age group, but the prevalence peaks across all age groups occurred between June and October.

### 3.2 Prevalence Surfaces From the DMAP Analysis

**Figure 4:**
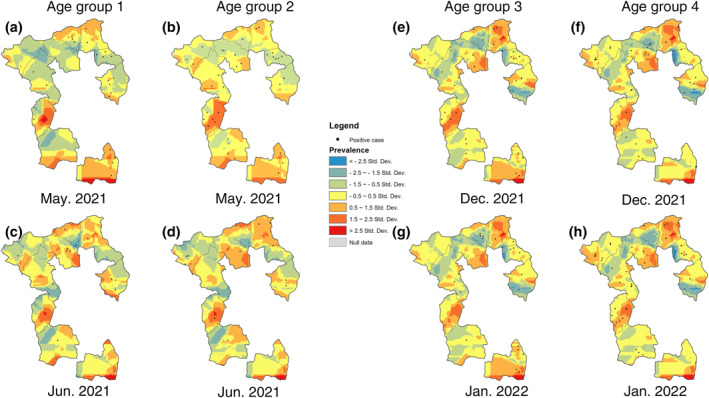
Smoothed prevalence surfaces of *Campylobacter* infections in different age groups by month in 10 kebeles of Ethiopia. Age Group 1: 0–95 days; age Group 2: 96–177 days; age Group 3: 178–285 days; age Group 4: >285 days. Those eight maps from 4 months were selected as representatives to demonstrate the spatial patterns of high/low‐prevalence areas. The full set of maps can be found in the Figure S1 of Supporting Information S1. Abbreviations: AW—Adele Walta; AM—Amuma; BK—Bachake; BG—Biftu Geda; DA—Damota; GC—Gobe Challa; IO—Ifa Oromia; KR—Kuro; NG—Nageya; QD—Qerensa Dereba. (a) Age group 1 in May 2021; (b) Age group 2 in May 2021; (c) Age group 1 in June 2021; (d) Age group 2 in June 2021; (e) Age group 3 in December 2021; (f) Age group 4 in December 2021; (g) Age group 3 in January 2022; (h) Age group 4 in January 2022.

**Figure 5:**
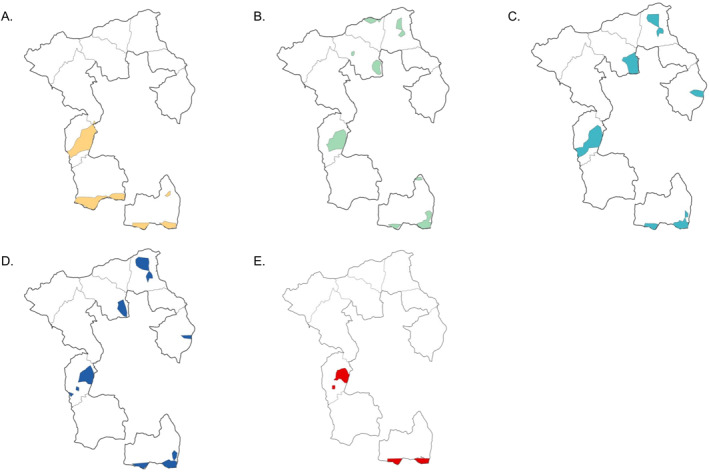
Distribution of areas with persistently high *Campylobacter* infection prevalence by age group in 10 kebeles of Ethiopia. Shaded areas with colors represent regions of persistently high *Campylobacter* prevalence. A—D: age groups 1–4; E: overlaps among four age groups. Abbreviations: AW—Adele Walta; AM—Amuma; BK—Bachake; BG—Biftu Geda; DA—Damota; GC—Gobe Challa; IO—Ifa Oromia; KR—Kuro; NG—Nageya; QD—Qerensa Dereba.

DMAP generated a series of smoothed prevalence surfaces for each age group by month. Spatially, areas with a higher prevalence of *Campylobacter* infections were distributed across the whole study area (Figure 4 and Figure S1 in Supporting Information S1). Four kebeles, Qerensa Dereba (QD) and Kuro (KR) in the north, Adele Walta (AW) in the southwest, and Nageya (NG) in the south, covered regions that had a relatively higher prevalence over time. Results from the STAMP analysis further demonstrated that these stable hot spots were distributed in central Qerensa Dereba (QD), the southeast of Kuro (KR), the south of Adele Walta (AW), as well as the southwest and southeast of Nageya (NG) (Figure 5). This spatial pattern tended to be similar among different age groups, with the exception that stable hot spots were only found in three kebeles in the south for age Group 1.

Meanwhile, several areas on the landscape had a constant lower prevalence than the average (less than 1.5 standard deviations). The prevalence in the southern part of Amuma (AM) has been persistently lower across the four age groups. The southeast corner of Ifa Oromia (IO) and the northwest of Gobe Challa (GC) had lower prevalence only in age Groups 1 and 2; as well as northern Ifa Oromia (IO), the west part of Bachake (BK), and the south part of Damota (DA) only in age Groups 3 and 4.

### 3.3 Ecological Niche Models

**Figure 6:**
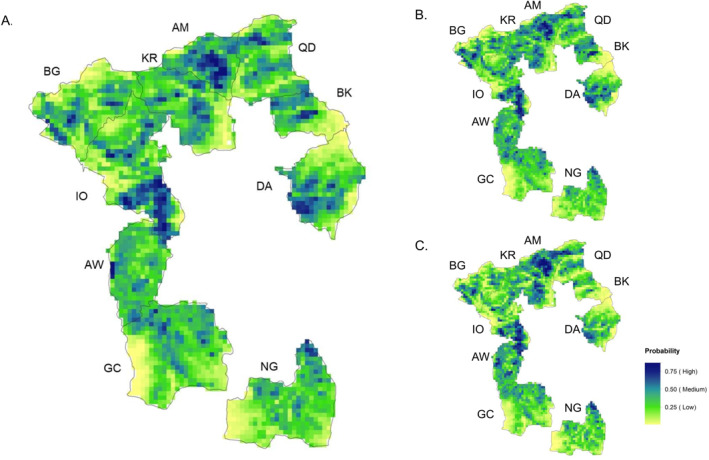
Habitat suitability maps for *Campylobacter* in 10 kebeles of Ethiopia. (a) genus *Campylobacter* (b) *C. jejuni* (c) *C. infans*. The values at each pixel were derived from the average of 10 bootstrapped replicates. Abbreviations: AW—Adele Walta; AM—Amuma; BK—Bachake; BG—Biftu Geda; DA—Damota; GC—Gobe Challa; IO—Ifa Oromia; KR—Kuro; NG—Nageya; QD—Qerensa Dereba.

For the genus‐level MaxEnt model, the average training AUC of 10 replicates was 0.841, with a standard deviation of 0.011 (Figure S2 in Supporting Information S1). The habitat suitability map indicated that Amuma (AM) and central Kuro (KR) in the north, the southern part of Ifa Oromia (IO) in the central west, and the southwest of Damota (DA) in the central east were highly suitable for the pathogen persistence compared to other regions (Figure 6a). There were also several areas with low habitat suitability. These were distributed in the southeastern Kuro (KR), adjacent areas of southeastern Bachake (BK) and east Damota (DA), and west parts of Gobe Challa (GC) and Nageya (NG).

*C. jejuni* and *C. infans* were recovered from environmental samples collected at 87 and 74 households, respectively. Both species were found to coexist in samples from 35 households. The suitability maps for the two dominant species (Figures 6b and 6c) showed similar patterns compared to the genus level. There was no apparent difference in the distribution of high/low suitable areas between these two species. Overall, kebeles in the north were predicted to be more suitable for the pathogen persistence than in the south. By overlaying the suitability maps to the smoothed prevalence surfaces generated from DMAP, we found that Qerensa Dereba (QD), part of Kuro (KR), and Adele Walta (AW) had both high habitat suitability and high prevalence of *Campylobacter* infection. Meanwhile, some areas, for example, southern parts of both Amuma (AM) and Damota (DA), had higher habitat suitability but lower prevalence.

The overall level of variation on the values at each pixel between 10 replicates of these three MaxEnt models was relatively low, with only a few regions showing a high standard deviation (Figure S3 in Supporting Information S1). That is, the habitat suitability in most regions of our study area was quite similar between the 10 bootstrap runs, suggesting relatively robust models obtained for the species distribution of *Campylobacter* in the study area.

Percent contribution and permutation importance are two measures of variable contributions in MaxEnt. Among the three MaxEnt models, elevation, mean 16‐day NDVI, and slope had the top 3 highest percent contribution (Table 2). However, the order of these three environmental covariates' permutation importance demonstrated a nuanced difference between models. In the genus model, the mean 16‐day NDVI had the highest permutation importance, followed by slope and elevation. While elevation had the highest value in the models for the two species. For the suitability of *C. jejuni*, slope became less important, and the minimum daily temperature was ranked the second highest after elevation. Similarly, the minimum daily temperature replaced the mean 16‐day NDVI and became more important in the *C. infans* model. For each environmental covariate in the MaxEnt model, a response curve was generated by plotting the predicted occurrence probability against the range of values of the corresponding variable. The range of the most suitable environmental conditions for the species can be identified through the response curves. The peaks in the response curves of the three most important contributors indicated that a moderate elevation of around 2100 m, a moderate mean NDVI value of around 0.40, and a gentle slope (∼5°) are highly suitable for the presence of *Campylobacter* at both the genus and species level (Figure S4 in Supporting Information S1).

**Table 2:** Percent Contribution and Permutation Importance of Environmental Variables in Three MaxEnt Models

Variable	Percent contribution			Permutation importance		
	Genus	*C. jejuni*	*C. infans*	Genus	*C. jejuni*	*C. infans*
Elevation	**19.9** ^a^	**24.1**	15.4	12.8	**19.3**	**18.9**
Mean 16‐day NDVI, 2021	14.5	16	15.5	**20.6**	14.7	8.2
Slope	12.4	17	**17.4**	16.7	9.3	14.5
Population count, 2020	9.6	6.2	10.5	3.2	4.4	7
Minimum daily land surface temperature, 2021	9.5	10.3	11.8	6.8	15.7	16.1
Mean daily land surface temperature, 2021	8.8	4.8	2.3	9	4.4	3.4
Maximum 16‐day NDVI, 2021	7.6	6.2	8.6	4.5	3.5	6.9
Maximum daily land surface temperature, 2021	5.6	4.5	2.9	6.5	6.2	1.1
Proportion of clay particles in the fine earth fraction	3.9	4	5.4	3.1	6.3	6.8
Soil organic carbon content in the fine earth fraction	2.6	1.1	1.5	3.7	4.2	2.5
Wind speed at 100 m above ground	2.6	1.7	2	8.2	7.2	5.7
Minimum 16‐day NDVI, 2021	2.4	3.6	5.8	3.2	3.7	6.7
Soil pH	0.6	0.6	1.1	1.6	1	2.2

^a^ Bold numbers represents the highest value in each column.

## 4 Discussion

Here, we estimated the prevalence of *Campylobacter* infection in a cohort of children from birth to 13 months of age in a rural part of eastern Ethiopia. We also mapped prevalence to evaluate spatial patterns of prevalence and used ecological niche modeling to predict environmental suitability for pathogen persistence using environmental covariates describing soil conditions, elevation, vegetation indices, and human population. Previously, the prevalence of *Campylobacter* infections (specifically *C. jejuni/coli*) in CU5 in Ethiopia was estimated at 14.5%, primarily based on studies of diarrheic children in hospitals or clinic centers (Gahamanyi et al., 2020). Given the high frequency of asymptomatic colonization of *Campylobacter* among young children in LMICs, the actual prevalence could be much higher than the estimate. The results from the formative research of the CAGED project in Ethiopia supported this assumption, showing a much higher prevalence of *Campylobacter* colonization than previous estimates—50% among children 11–13 months of age when those with or without symptoms are included (Chen, McKune, et al., 2021). When examining each individual age group identified by *K*‐means clustering, we observed a clear upward trend of the prevalence among infants as they grew older, which is consistent with the findings from the MAL‐ED study (Amour et al., 2016). As documented in detail elsewhere (Deblais et al., 2023), we observed that prevalence reached 80%–100% for children older than 285 days. Peaks of prevalence across all age groups occurred between June and October during the study period.

The generation of smoothed prevalence surfaces by age group and month suggests that *Campylobacter* is ubiquitous in this relatively small region, both spatially and temporally. However, that doesn't necessarily imply a uniform spatial distribution of infections with this pathogen. Local areas with persistently higher/lower prevalence over time were identified in all age groups. High‐prevalence areas were found in the north, southwest, and south of the study region, and these areas overlapped more or less through all four age groups. Several regions with constantly low prevalence were distributed adjacent to these high‐prevalence areas in the north and central areas. What environmental/non‐environmental factors contribute to this pattern is beyond the scope of this study and needs further investigation.

In this study, we first examined the habitat suitability for *Campylobacter* at the genus level and then for two dominant species over the study area using MaxEnt models with environmental covariates. The MaxEnt models in this study provided a robust prediction of *Campylobacter* distribution, and the suitability maps predicted by the MaxEnt models confirmed the environmental signal suggested by the DMAP analysis ‐ *Campylobacter* is ubiquitous but not uniformly distributed. Due to this ubiquitousness of *Campylobacter* species across the landscape, the suitability maps from both genus and species‐specific models did not show any apparent differences in the distribution of high/low suitable areas. Environmental covariates including elevation, slope, and NDVI contributed to predicting the distribution of *Campylobacter* genus and species. On the other hand, we also noted that some areas, for example, southern parts of both Amuma (AM) and Damota (DA), had higher habitat suitability for *Campylobacter* but a lower prevalence of *Campylobacter* infection after comparing the suitability map with the smoothed prevalence surfaces. Since ecological niche models only tackle the ecological covariates or abiotic signals (Brookes et al., 2020), this suggests that potentially biotic factors, for example, interactions between infants and other human or livestock reservoirs of the pathogen, may affect the distribution of these bacteria in the study area.

It is important to note that environmental samples, including drinking water, soil, and livestock feces, were used in this study solely to inform ecological niche models predicting the environmental suitability for *Campylobacter* presence. These models do not establish direct epidemiological links or confirm transmission routes from environmental sources to children. As such, while the ecological patterns observed suggest that environmental conditions may contribute to shaping the distribution of *Campylobacter* in this setting, further studies are needed to establish causal relationships between environmental conditions and *Campylobacter* carriage in children. Specifically, integrating molecular typing or whole‐genome sequencing of isolates from both human and environmental samples would provide more definitive evidence of transmission pathways. Notably, another study from our team, which applied multilocus sequence typing (MLST), has been published and explores the transmission dynamics of *Campylobacter* between infants and environmental sources in this population (Singh et al., 2025). That work provides complementary strain‐level evidence to support the hypotheses generated from the current ecological modeling approach.

In addition, we acknowledge that PCR‐based detection in environmental samples does not distinguish between live and dead bacteria, and therefore, the presence of *Campylobacter* DNA may not always reflect viable organisms capable of transmission. While our ecological niche modeling approach relies on presence/absence data to identify areas of potential environmental suitability, we recognize that this method does not confirm exposure risk or viability.

Unlike in HICs where the sources of *Campylobacter* infections are frequently linked to the consumption of contaminated food, the risk factors for *Campylobacter*iosis in LMICs are more associated with the environmental pathways, for example, contaminated drinking water and soil, and direct contact with domestic animals (Budge et al., 2020; Coker et al., 2002; Schiaffino et al., 2021). Multilevel factors are involved in these pathways. At the regional level, climatic covariates could be drivers in spreading the pathogen in the environment. For example, runoff formed during the rainy season, along with rainfall itself, might lead to the contamination of drinking water and potentially facilitate the dispersion of *Campylobacter*. Meanwhile, household‐level variables may play a more direct role in influencing the interaction between children and *Campylobacter* (Chen, Mechlowitz, et al., 2021). For example, the practice of livestock husbandry on or in the homestead could increase children's exposure to the pathogen through contacting animal feces or soil contaminated by animal feces. Thus, factors at both levels should be considered when further explaining the spatial pattern of *Campylobacter* infections.

Due to the relative sparseness of data points for the two dominant species compared to the genus level, we did not perform DMAP analysis on these two species to examine the monthly patterns of infections among children. This was a limitation in our study. Though suitability maps suggested no difference between both levels, it is still worthwhile to characterize infections with these two dominant species among young children in the study area and determine their temporal patterns in future studies.

Hot spots of *Campylobacter* infections identified through the DMAP and STAMP analyses could be targeting areas for future interventions, especially those areas with a persistently high prevalence. MaxEnt‐based distribution map has suggested that environmental covariates, including elevation, slope, and the mean NDVI, were major factors influencing the distribution of *Campylobacter* at both genus and species levels. It also demonstrated areas that are more likely to support the distribution of *Campylobacter* and should be emphasized in future *Campylobacter* surveillance programs in the study area. Though not addressed in this study, identifying potential factors that contribute to low prevalence in highly suitable areas for *Campylobacter* will be worthwhile in future studies, and relevant results will guide the development of effective intervention strategies for protecting children from *Campylobacter* infections and associated long‐term health effects.

## Conflict of Interest

The authors declare no conflicts of interest relevant to this study.

## Supporting information

Supporting Information S1

Movie S1

Movie S2

Movie S3

Movie S4

## Data Availability

The data on which this article is based are available in Havelaar (2025).

## References

[gh270123-bib-0001] Alexander, K. A. , Lewis, B. L. , Marathe, M. , Eubank, S. , & Blackburn, J. K. (2012). Modeling of wildlife‐associated zoonoses: Applications and caveats. Vector Borne and Zoonotic Diseases, 12(12), 1005–1018. 10.1089/vbz.2012.0987 23199265 PMC3525896

[gh270123-bib-0002] Amour, C. , Gratz, J. , Mduma, E. R. , Svensen, E. , Rogawski, E. T. , McGrath, M. , et al. (2016). Epidemiology and impact of campylobacter infection in children in 8 low‐resource settings: Results from the MAL‐ED study. Clinical Infectious Diseases: An Official Publication of the Infectious Diseases Society of America, 63(9), 1171–1179. 10.1093/CID/CIW542 27501842 PMC5064165

[gh270123-bib-0003] Arsenault, J. , Berke, O. , Michel, P. , Ravel, A. , & Gosselin, P. (2012). Environmental and demographic risk factors for campylobacteriosis: Do various geographical scales tell the same story? BMC Infectious Diseases, 12(1), 1–12. 10.1186/1471-2334-12-318/FIGURES/2 23173982 PMC3570353

[gh270123-bib-0004] Bartlow, A. W. , Giermakowski, J. T. , Painter, C. W. , Neville, P. , Schultz‐Fellenz, E. S. , Crawford, B. M. , et al. (2022). Modeling the distribution of the endangered Jemez Mountains salamander (Plethodon neomexicanus) in relation to geology, topography, and climate. Ecology and Evolution, 12(8), e9161. 10.1002/ECE3.9161 36035267 PMC9399451

[gh270123-bib-0005] Bian, X. , Garber, J. M. , Cooper, K. K. , Huynh, S. , Jones, J. , Mills, M. K. , et al. (2020). Campylobacter abundance in breastfed infants and identification of a new species in the global enterics multicenter study. mSphere, 5(1), e00735‐19. 10.1128/msphere.00735-19 31941810 PMC6968651

[gh270123-bib-0006] Blackburn, J. K. (2010). Integrating geographic information systems and ecological niche modeling into disease ecology: A case study of bacillus anthracis in the United States and Mexico. In K. O'Connell , E. Skowronski , A. Sulakvelidze , & L. Bakanidze (Eds.), Emerging and endemic pathogens. NATO science for peace and security series A: Chemistry and biology. Springer Netherlands. 10.1007/978-90-481-9637-1_7

[gh270123-bib-0007] Brookes, V. J. , Belkhiria, J. A. , Machado, G. , & Escobar, L. E. (2020). Ecological niche modeling: An introduction for veterinarians and epidemiologists. Review, 7, 519059. 10.3389/fvets.2020.519059 PMC7641643 33195507

[gh270123-bib-0008] Budge, S. , Barnett, M. , Hutchings, P. , Parker, A. , Tyrrel, S. , Hassard, F. , et al. (2020). Risk factors and transmission pathways associated with infant Campylobacter spp. prevalence and malnutrition: A formative study in rural Ethiopia. PLoS One, 15(5), 1–17. 10.1371/journal.pone.0232541 PMC7209302 32384130

[gh270123-bib-0009] Chen, D. , McKune, S. , Yang, Y. , Usmane, I. A. , Ahmed, I. A. , Amin, J. K. , et al. (2024). Campylobacter colonization and undernutrition in infants in rural Eastern Ethiopia: A longitudinal community‐based birth cohort study. Frontiers in Public Health, 12, 1467462. 10.3389/FPUBH.2024.1467462 39839388 PMC11747651

[gh270123-bib-0010] Chen, D. , McKune, S. L. , Singh, N. , Yousuf Hassen, J. , Gebreyes, W. , Manary, M. J. , et al. (2021). Campylobacter colonization, environmental enteric dysfunction, stunting, and associated risk factors among young children in rural Ethiopia: A cross‐sectional study from the campylobacter genomics and environmental enteric dysfunction (CAGED) project. Frontiers in Public Health, 8, 1043. 10.3389/fpubh.2020.615793 PMC7862945 33553097

[gh270123-bib-0011] Chen, D. , Mechlowitz, K. , Li, X. , Schaefer, N. , Havelaar, A. H. , & McKune, S. L. (2021). Benefits and risks of smallholder livestock production on child nutrition in low‐ and middle‐income countries. Frontiers in Nutrition, 8, 751686. 10.3389/FNUT.2021.751686 34778344 PMC8579112

[gh270123-bib-0012] Coker, A. O. , Isokpehi, R. D. , Thomas, B. N. , Amisu, K. O. , & Larry Obi, C. (2002). Human campylobacteriosis in developing countries. Emerging Infectious Diseases, 8(3), 237–243. 10.3201/eid0803.010233 11927019 PMC2732465

[gh270123-bib-0013] Colston, J. M. , Zaitchik, B. F. , Badr, H. S. , Burnett, E. , Ali, S. A. , Rayamajhi, A. , et al. (2022). Associations between eight Earth observation‐derived climate variables and enteropathogen infection: An independent participant data meta‐analysis of surveillance studies with broad spectrum nucleic acid diagnostics. GeoHealth, 6(1), e2021GH000452. 10.1029/2021GH000452 PMC8729196 35024531

[gh270123-bib-0014] Deblais, L. , Ojeda, A. , Brhane, M. , Mummed, B. , Hassen, K. A. , Ahmedo, B. U. , et al. (2023). Prevalence and load of the campylobacter genus in infants and associated household contacts in rural eastern Ethiopia: A longitudinal study from the campylobacter genomics and environmental enteric dysfunction (CAGED) project. Applied and Environmental Microbiology, 89(7), e00424‐23. 10.1128/aem.00424-23 37310259 PMC10370295

[gh270123-bib-0015] Elith, J. , Graham, C. H. , P. Anderson, R. , Dudík, M. , Ferrier, S. , Guisan, A. , et al. (2006). Novel methods improve prediction of species' distributions from occurrence data. Ecography. 10.1111/j.2006.0906-7590.04596.x

[gh270123-bib-0016] Elith, J. , & Leathwick, J. R. (2009). Species distribution models: Ecological explanation and prediction across space and time. Annual Review of Ecology Evolution and Systematics, 40(1), 677–697. 10.1146/annurev.ecolsys.110308.120159

[gh270123-bib-0017] Elith, J. , Phillips, S. J. , Hastie, T. , Dudík, M. , Chee, Y. E. , & Yates, C. J. (2011). A statistical explanation of MaxEnt for ecologists. Diversity and Distributions, 17(1), 43–57. 10.1111/J.1472-4642.2010.00725.X

[gh270123-bib-0018] El‐Tras, W. F. , Holt, H. R. , Tayel, A. A. , & El‐Kady, N. N. (2015). Campylobacter infections in children exposed to infected backyard poultry in Egypt. Epidemiology and Infection, 143(2), 308–315. 10.1017/S095026881400096X 24774694 PMC9206777

[gh270123-bib-0019] François, R. , Yori, P. P. , Rouhani, S. , Siguas Salas, M. , Paredes Olortegui, M. , Rengifo Trigoso, D. , et al. (2018). The other campylobacters: Not innocent bystanders in endemic diarrhea and dysentery in children in low‐income settings. PLoS Neglected Tropical Diseases, 12(2), e0006200. 10.1371/journal.pntd.0006200 29415075 PMC5819825

[gh270123-bib-0020] Gahamanyi, N. , Mboera, L. E. G. , Matee, M. I. , Mutangana, D. , & Komba, E. V. G. (2020). Prevalence, risk factors, and antimicrobial resistance profiles of thermophilic campylobacter species in humans and animals in Sub‐Saharan Africa: A systematic review. International Journal of Microbiology, 2020, 1–12. 10.1155/2020/2092478 PMC6983289 32025233

[gh270123-bib-0021] Garcia Bardales, P. F. , Schiaffino, F. , Huynh, S. , Olortegui, M. P. , Yori, P. P. , Vasquez, T. P. , et al. (2022). “Candidatus Campylobacter infans” detection is not associated with diarrhea in children under the age of 2 in Peru. PLoS Neglected Tropical Diseases, 16(10), 1–12. 10.1371/JOURNAL.PNTD.0010869 PMC9612815 36251729

[gh270123-bib-0022] Gorris, M. E. , Bartlow, A. W. , Temple, S. D. , Romero‐Alvarez, D. , Shutt, D. P. , Fair, J. M. , et al. (2021). Updated distribution maps of predominant Culex mosquitoes across the Americas. Parasites & Vectors, 14(1), 547. 10.1186/s13071-021-05051-3 34688314 PMC8542338

[gh270123-bib-0023] Haque, M. A. , Platts‐Mills, J. A. , Mduma, E. , Bodhidatta, L. , Bessong, P. , Shakoor, S. , et al. (2019). Determinants of Campylobacter infection and association with growth and enteric inflammation in children under 2 years of age in low‐resource settings. Scientific Reports, 9(1), 17124. 10.1038/S41598-019-53533-3 31748573 PMC6868199

[gh270123-bib-0024] Hartigan, J. A. , & Wong, M. A. (1979). Algorithm AS 136: A K‐Means clustering algorithm. Journal of the Royal Statistical Society. Series c (Applied Statistics), 28(1), 100–108. 10.2307/2346830

[gh270123-bib-0025] Havelaar, A. (2025). Longitudinal data on campylobacter in infants from the CAGED study [Dataset]. Harvard Dataverse. 10.7910/DVN/1JC6RS

[gh270123-bib-0026] Havelaar, A. H. , Brhane, M. , Ahmed, I. A. , Kedir, J. , Chen, D. , Deblais, L. , et al. (2022). Unravelling the reservoirs for colonisation of infants with Campylobacter spp. in rural Ethiopia: Protocol for a longitudinal study during a global pandemic and political tensions. BMJ Open, 12(10), 61311. 10.1136/bmjopen-2022-061311 PMC9535169 36198455

[gh270123-bib-0027] Havelaar, A. H. , Kirk, M. D. , Torgerson, P. R. , Gibb, H. J. , Hald, T. , Lake, R. J. , et al. (2015). World health organization global estimates and regional comparisons of the burden of foodborne disease in 2010. PLoS Medicine, 12(12), e1001923. 10.1371/journal.pmed.1001923 26633896 PMC4668832

[gh270123-bib-0028] Joyner, T. A. , Lukhnova, L. , Pazilov, Y. , Temiralyeva, G. , Hugh‐Jones, M. E. , Aikimbayev, A. , & Blackburn, J. K. (2010). Modeling the potential distribution of Bacillus anthracis under multiple climate change scenarios for Kazakhstan. PLoS One, 5(3), e9596. 10.1371/journal.pone.0009596 20231894 PMC2834750

[gh270123-bib-0029] Kaakoush, N. O. , Castaño‐Rodríguez, N. , Mitchell, H. M. , & Man, S. M. (2015). Global epidemiology of campylobacter infection. Clinical Microbiology Reviews, 28(3), 687–720. 10.1128/CMR.00006-15 26062576 PMC4462680

[gh270123-bib-0030] Keusch, G. T. , Denno, D. M. , Black, R. E. , Duggan, C. , Guerrant, R. L. , Lavery, J. V. , et al. (2014). Environmental enteric dysfunction: Pathogenesis, diagnosis, and clinical consequences. Clinical Infectious Diseases, 59(suppl_4), S207–S212. 10.1093/cid/ciu485 25305288 PMC4481570

[gh270123-bib-0031] Kuhn, K. G. , Nygård, K. M. , Guzman‐Herrador, B. , Sunde, L. S. , Rimhanen‐Finne, R. , Trönnberg, L. , et al. (2020). Campylobacter infections expected to increase due to climate change in Northern Europe. Scientific Reports, 10(1), 1–9. 10.1038/s41598-020-70593-y 32807810 PMC7431569

[gh270123-bib-0032] Kumar, A. , Kumar, A. , Adhikari, D. , Gudasalamani, R. , Saikia, P. , & Khan, M. L. (2020). Ecological niche modeling for assessing potential distribution of Pterocarpus marsupium Roxb. In Ranchi, eastern India. Ecological Research. 10.1111/1440-1703.12176

[gh270123-bib-0033] Lee, G. , Pan, W. , Peñataro Yori, P. , Paredes Olortegui, M. , Tilley, D. , Gregory, M. , et al. (2013). Symptomatic and asymptomatic campylobacter infections associated with reduced growth in Peruvian children. PLoS Neglected Tropical Diseases, 7(1), e2036. 10.1371/journal.pntd.0002036 23383356 PMC3561130

[gh270123-bib-0034] Lengerh, A. , Moges, F. , Unakal, C. , & Anagaw, B. (2013). Prevalence, associated risk factors and antimicrobial susceptibility pattern of Campylobacter species among under five diarrheic children at Gondar University Hospital, Northwest Ethiopia. BMC Pediatrics, 13(1), 82. 10.1186/1471-2431-13-82 23694714 PMC3663702

[gh270123-bib-0035] Lentz, J. A. , Blackburn, J. K. , & Curtis, A. J. (2011). Evaluating patterns of a white‐band disease (WBD) outbreak in acropora palmata using spatial analysis: A comparison of transect and colony clustering. PLoS One, 6(7), e21830. 10.1371/journal.pone.0021830 21818271 PMC3139597

[gh270123-bib-0036] Long, J. , Robertson, C. , & Nelson, T. (2018). Stampr: Spatial‐temporal analysis of moving polygons in R. Journal of Statistical Software, 84(April). 10.18637/jss.v084.c01

[gh270123-bib-0037] Louis, V. R. , Gillespie, I. A. , O’brien, S. J. , Russek‐Cohen, E. , Pearson, A. D. , & Colwell, R. R. (2005). Temperature‐driven campylobacter seasonality in England and Wales. Applied and Environmental Microbiology, 71(1), 85–92. 10.1128/AEM.71.1.85-92.2005 15640174 PMC544220

[gh270123-bib-0038] Luby, S. P. , Rahman, M. , Arnold, B. F. , Unicomb, L. , Ashraf, S. , Winch, P. J. , et al. (2018). Effects of water quality, sanitation, handwashing, and nutritional interventions on diarrhoea and child growth in rural Bangladesh: A cluster randomised controlled trial. Lancet Global Health, 6(3), e302–e315. 10.1016/S2214-109X(17)30490-4 29396217 PMC5809718

[gh270123-bib-0039] Man, S. M. (2011). The clinical importance of emerging Campylobacter species. Nature Reviews Gastroenterology & Hepatology, 8(12), 669–685. 10.1038/nrgastro.2011.191 22025030

[gh270123-bib-0040] McQuade, E. T. R. , Platts‐Mills, J. A. , Gratz, J. , Zhang, J. , Moulton, L. H. , Mutasa, K. , et al. (2020). Impact of water quality, sanitation, handwashing, and nutritional interventions on enteric infections in rural Zimbabwe: The Sanitation Hygiene Infant Nutrition Efficacy (SHINE) trial. The Journal of Infectious Diseases, 221(8), 1379–1386. 10.1093/INFDIS/JIZ179 31004129 PMC7325799

[gh270123-bib-0041] Merow, C. , Smith, M. J. , & Silander, J. A. (2013). A practical guide to MaxEnt for modeling species’ distributions: What it does, and why inputs and settings matter. Ecography, 36(10), 1058–1069. 10.1111/J.1600-0587.2013.07872.X

[gh270123-bib-0042] Mullins, J. , Lukhnova, L. , Aikimbayev, A. , Pazilov, Y. , Ert, M. V. , & Blackburn, J. K. (2011). Ecological Niche modelling of the *Bacillus anthracis* A1.a sub‐lineage in Kazakhstan. BMC Ecology, 11(1), 32. 10.1186/1472-6785-11-32 22152056 PMC3260114

[gh270123-bib-0043] Null, C. , Stewart, C. P. , Pickering, A. J. , Dentz, H. N. , Arnold, B. F. , Arnold, C. D. , et al. (2018). Effects of water quality, sanitation, handwashing, and nutritional interventions on diarrhoea and child growth in rural Kenya: A cluster‐randomised controlled trial. Lancet Global Health, 6(3), e316–e329. 10.1016/S2214-109X(18)30005-6 29396219 PMC5809717

[gh270123-bib-0044] Ojeda, A. , Deblais, L. , Mummed, B. , Brhane, M. , Hassen, K. A. , Ahmedo, B. U. , et al. (2025). Determinants of Campylobacter species diversity in infants and association with family members, livestock, and household environments in rural Eastern Ethiopia. Gut Pathogens, 17(1), 1–15. 10.1186/S13099-025-00725-0 40618155 PMC12228300

[gh270123-bib-0045] Parker, C. T. , Schiaffino, F. , Huynh, S. , Paredes Olortegui, M. , Peñataro Yori, P. , Garcia Bardales, P. F. , et al. (2022). Shotgun metagenomics of fecal samples from children in Peru reveals frequent complex co‐infections with multiple Campylobacter species. PLoS Neglected Tropical Diseases, 16(10), 1–17. 10.1371/journal.pntd.0010815 PMC9565744 36194603

[gh270123-bib-0046] Philips, S. J. , Dudik, M. , & Schapire, R. E. (n.d.). Maxent software for modeling species niches and distributions (Version 3.4.4). Retrieved from http://biodiversityinformatics.amnh.org/open_source/maxent/

[gh270123-bib-0047] Phillips, S. J. (2021). A brief tutorial on Maxent. Retrieved from https://biodiversityinformatics.amnh.org/open_source/maxent/Maxent_tutorial_2021.pdf

[gh270123-bib-0048] Phillips, S. J. , Anderson, R. P. , & Schapire, R. E. (2006). Maximum entropy modeling of species geographic distributions. Ecological Modelling, 190(3–4), 231–259. 10.1016/j.ecolmodel.2005.03.026

[gh270123-bib-0049] Platts‐Mills, J. A. , & Kosek, M. (2014). Update on the burden of Campylobacter in developing countries. Current Opinion in Infectious Diseases, 27(5), 444–450. 10.1097/QCO.0000000000000091 25023741 PMC4542018

[gh270123-bib-0050] Platts‐Mills, J. A. , Liu, J. , Gratz, J. , Mduma, E. , Amour, C. , Swai, N. , et al. (2014). Detection of Campylobacter in stool and determination of significance by culture, enzyme immunoassay, and PCR in developing countries. Journal of Clinical Microbiology, 52(4), 1074–1080. 10.1128/JCM.02935-13 24452175 PMC3993515

[gh270123-bib-0051] Reiner, R. C. , Wiens, K. E. , Deshpande, A. , Baumann, M. M. , Lindstedt, P. A. , Blacker, B. F. , et al. (2020). Mapping geographical inequalities in childhood diarrhoeal morbidity and mortality in low‐income and middle‐income countries, 2000‐17: Analysis for the Global Burden of Disease Study 2017. The Lancet, 395(10239), 1779–1801. 10.1016/S0140-6736(20)30114-8 PMC7314599 32513411

[gh270123-bib-0052] Robertson, C. , Nelson, T. A. , Boots, B. , & Wulder, M. A. (2007). STAMP: Spatial‐temporal analysis of moving polygons. Journal of Geographical Systems, 9(3), 207–227. 10.1007/s10109-007-0044-2

[gh270123-bib-0053] Rogawski, E. T. , Liu, J. , Platts‐Mills, J. A. , Kabir, F. , Lertsethtakarn, P. , Siguas, M. , et al. (2018). Use of quantitative molecular diagnostic methods to investigate the effect of enteropathogen infections on linear growth in children in low‐resource settings: Longitudinal analysis of results from the MAL‐ED cohort study. Lancet Global Health, 6(12), e1319–e1328. 10.1016/S2214-109X(18)30351-6 30287125 PMC6227248

[gh270123-bib-0054] Rushton, G. , & Lolonis, P. (1996). Exploratory spatial analysis of birth defect rates in an urban population. In Statistics in medicine. 10.1002/(SICI)1097-0258(19960415)15:7/9<717::AID-SIM243>3.0.CO;2-0 9132899

[gh270123-bib-0055] Sanderson, R. A. , Maas, J. A. , Blain, A. P. , Gorton, R. , Ward, J. , O’Brien, S. J. , et al. (2018). Spatio‐temporal models to determine association between Campylobacter cases and environment. International Journal of Epidemiology, 47(1), 202–216. 10.1093/ije/dyx217 29069406 PMC5837245

[gh270123-bib-0056] Schiaffino, F. , Trigoso, D. R. , Colston, J. M. , Olortegui, M. P. , Shapiama Lopez, W. V. , Garcia Bardales, P. F. , et al. (2021). Associations among household animal ownership, infrastructure, and hygiene characteristics with source attribution of household fecal contamination in Peri‐urban communities of Iquitos, Peru. The American Journal of Tropical Medicine and Hygiene, 104(1), 372–381. 10.4269/AJTMH.20-0810 33146117 PMC7790101

[gh270123-bib-0057] Singh, N. , Thystrup, C. A. N. , Hassen, B. M. , Bhandari, M. , Rajashekara, G. , Hald, T. M. , et al. (2025). Transmission pathways of Campylobacter Jejuni between humans and livestock in rural Ethiopia are highly complex and interdependent. Gut Pathogens, 17(1), 1–16. 10.1186/s13099-025-00691-7 40319305 PMC12049777

[gh270123-bib-0058] Soneja, S. , Jiang, C. , Romeo Upperman, C. , Murtugudde, R. , Mitchell, C. S. , Blythe, D. , et al. (2016). Extreme precipitation events and increased risk of campylobacteriosis in Maryland, U.S.A. Environmental Research, 149, 216–221. 10.1016/J.ENVRES.2016.05.021 27214137

[gh270123-bib-0059] Stewart, C. P. , Kariger, P. , Fernald, L. , Pickering, A. J. , Arnold, C. D. , Arnold, B. F. , et al. (2018). Effects of water quality, sanitation, handwashing, and nutritional interventions on child development in rural Kenya (WASH benefits Kenya): A cluster‐randomised controlled trial. The Lancet Child & Adolescent Health, 2(4), 269–280. 10.1016/S2352-4642(18)30025-7 29616236 PMC5859215

[gh270123-bib-0060] Terefe, Y. , Deblais, L. , Ghanem, M. , Helmy, Y. A. , Mummed, B. , Chen, D. , et al. (2020). Co‐occurrence of campylobacter species in children from Eastern Ethiopia, and their Association with environmental enteric dysfunction, diarrhea, and host microbiome. Frontiers in Public Health, 8, 99. 10.3389/fpubh.2020.00099 32351922 PMC7174729

[gh270123-bib-0061] Weisent, J. , Seaver, W. , Odoi, A. , & Rohrbach, B. (2014). The importance of climatic factors and outliers in predicting regional monthly campylobacteriosis risk in Georgia, USA. International Journal of Biometeorology, 58(9), 1865–1878. 10.1007/S00484-014-0788-6 24458769 PMC4190453

