# Supplementary material for: Spatial and Temporal Patterns of Campylobacter Infection and Projected Habitat Suitability of Dominant Campylobacter Species in Eastern Ethiopia
Source: Geohealth. 2026 Apr 10;10(4):e2024GH001146. doi: 10.1029/2024GH001146 (PMC13066913; doi:10.1029/2024GH001146)
Supplement: Supplementary file 1 — Supporting Information S1 [file GH2-10-e2024GH001146-s003.pdf]

GeoHealth

Supporting Information for

Spatial and Temporal Patterns of *Campylobacter* Infection and Projected Habitat Suitability of Dominant *Campylobacter* Species in Eastern Ethiopia

Xiaolong Li[1,2], Amanda E. Ojeda[3], Loic Deblais[4], Bahar Mummed Hassen[5], Mussie Bhrane[5], Gireesh Rajashekara[4], Song Liang[1,2], Jemal Yousuf Hassen[5], Sarah L. McKune[1,6,7], Arie H. Havelaar[2,3,7], Jason K. Blackburn[2,8] on behalf of the CAGED Research Team

[1]Department of Environmental and Global Health, College of Public Health and Health Professions, University of Florida, Gainesville, FL, USA

[2]Emerging Pathogens Institute, University of Florida, Gainesville, FL, USA

[3]Department of Microbiology and Cell Science, Institute of Food and Agricultural Sciences, University of Florida, Gainesville, FL, USA

[4]Center for Food Animal Health, Department of Animal Sciences, College of Food, Agricultural, and Environmental Sciences, The Ohio State University, Wooster, OH, USA

[5]Haramaya University, Haramaya, Oromia Regional State, Ethiopia

[6]Center for African Studies, University of Florida, Gainesville, FL, USA

[7]Global Food Systems Institute, University of Florida, Gainesville, FL, USA

[8]Spatial Epidemiology and Ecology Research Laboratory, Department of Geography, University of Florida, Gainesville, FL, USA

Contents of this file

Figures S1 to S3

Additional Supporting Information (Files uploaded separately)

Captions for Movies S1 to S4

Introduction

The supporting information includes three figures that provide additional information for the results of spatial filtering analysis and of ecological niche modeling in the main text. Spatial filtering analysis generated a series of smoothed prevalence maps of *Campylobacter* infections for different age groups by month. Of them, eight representative maps were selected to present the major patterns in the main text (Figure 4), and the full set of maps for each age group were combined into an animation GIF file as Movies S1 to S4.

For the MaxEnt models, we randomly split the input dataset into 80% for training and 20% for testing and ran 10 replicates with a bootstrapping procedure to account for the variations between models using different sets of training/test points. Model performance was evaluated by the area under the receiver operating curve (AUC). Figure S2 contains the mean AUC curves of ten replicated MaxEnt models. The average of these 10 bootstrapped models was then used to estimate the habitat suitability of *Campylobacter* genus and each of the two species on the landscape. The uncertainty of our models was presented by the standard deviation of habitat suitability at each pixel for the 10 replicates as presented in Figure S3.

For each environmental covariate in the MaxEnt model, a response curve was generated by plotting the predicted occurrence probability against the range of values of the corresponding variable. The range of the most suitable environmental conditions for the species can be identified through the response curves. Figure S4 includes the response curves of three most important environmental covariates (elevation, NDVI, and slope) in the MaxEnt models.

Movie S1. Smoothed prevalence surfaces of *Campylobacter* infections among infants in 10 kebeles of Ethiopia during December 2020 to June 2022 for Age group 1(0 – 95days).

Movie S2. Smoothed prevalence surfaces of *Campylobacter* infections among infants in 10 kebeles of Ethiopia during December 2020 to June 2022 for Age group 2 (96 – 177 days).

Movie S3. Smoothed prevalence surfaces of *Campylobacter* infections among infants in 10 kebeles of Ethiopia during December 2020 to June 2022 for Age group 3 (178 – 285 days).

Movie S4. Smoothed prevalence surfaces of *Campylobacter* infections among infants in 10 kebeles of Ethiopia during December 2020 to June 2022 for Age group 4 (> 285 days).

A.

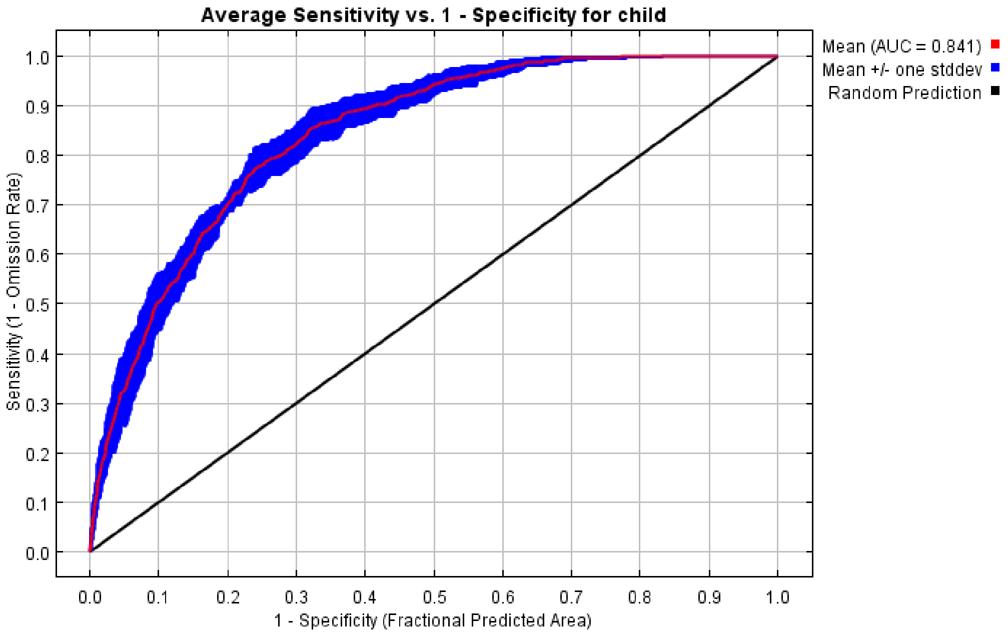
Average Sensitivity vs. 1 - Specificity for child
Sensitivity (1 - Omission Rate)
1 - Specificity (Fractional Predicted Area)
Mean (AUC = 0.841)
Mean +/- one stddev
Random Prediction
0.0
0.1
0.2
0.3
0.4
0.5
0.6
0.7
0.8
0.9
1.0

B.

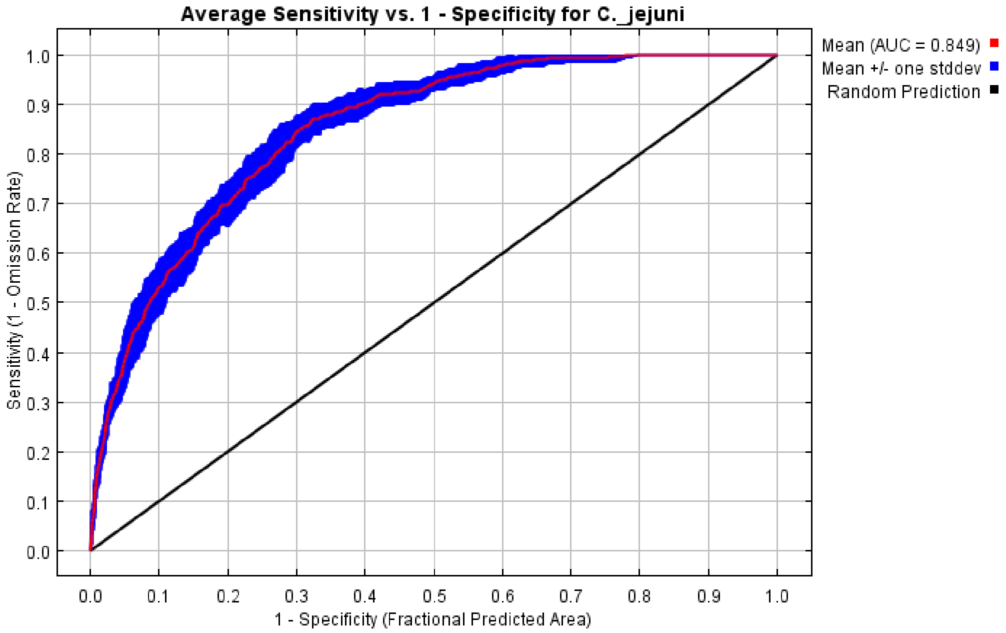
Average Sensitivity vs. 1 - Specificity for C._jejuni
Sensitivity (1 - Omission Rate)
1 - Specificity (Fractional Predicted Area)
Mean (AUC = 0.849)
Mean +/- one stddev
Random Prediction
0.0
0.1
0.2
0.3
0.4
0.5
0.6
0.7
0.8
0.9
1.0

C.

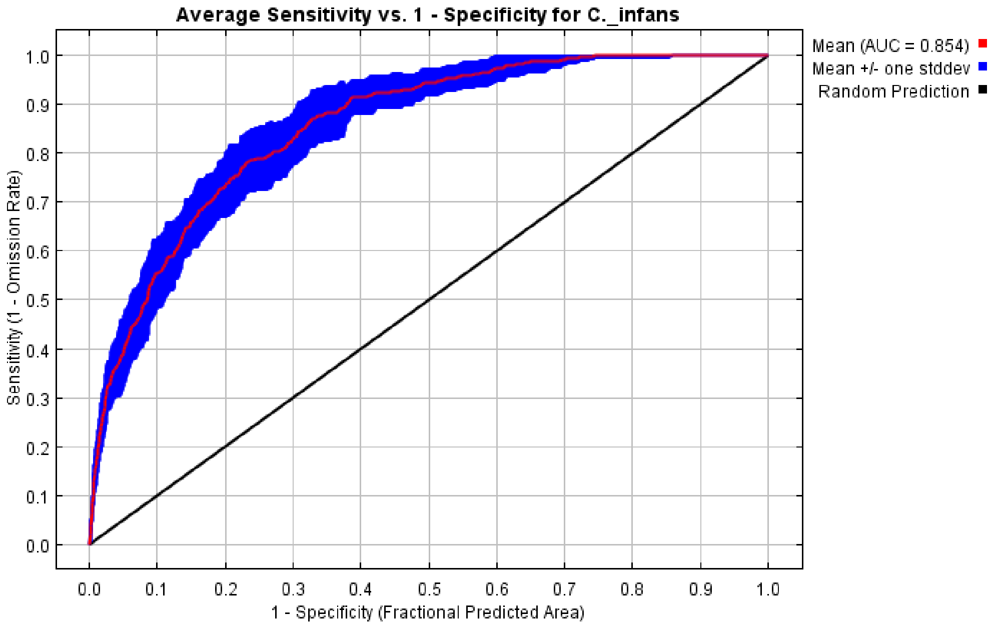

Figure S1. Mean AUC curve of ten replicated MaxEnt models. A. genus level; B. *Campylobacter jejuni*; C. *Campylobacter infans.*

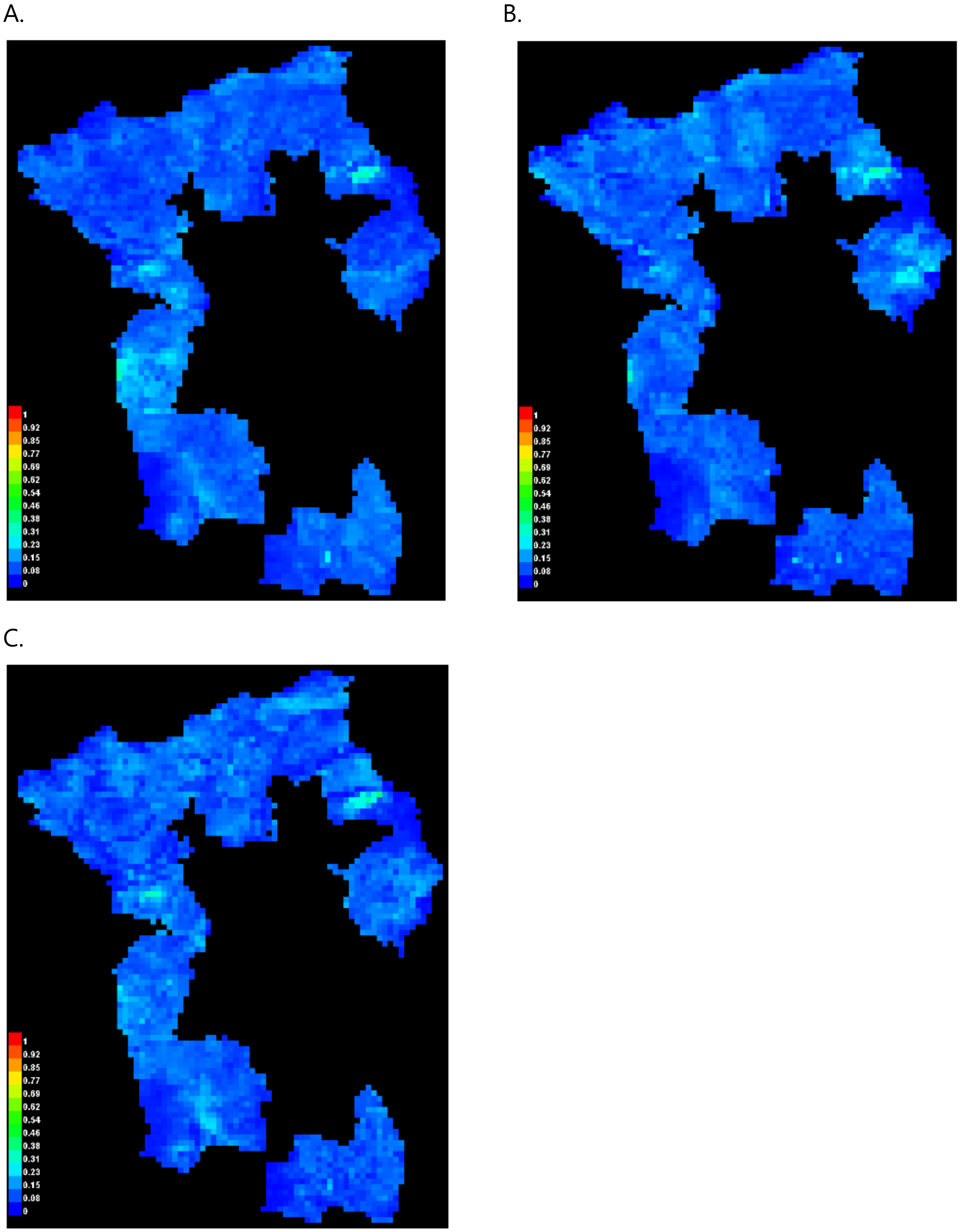

Figure S2. Standard deviation of habitat suitability at each pixel for the 10 replicated MaxEnt models. A. genus level; B. *Campylobacter jejuni*; C. *Campylobacter infans*.

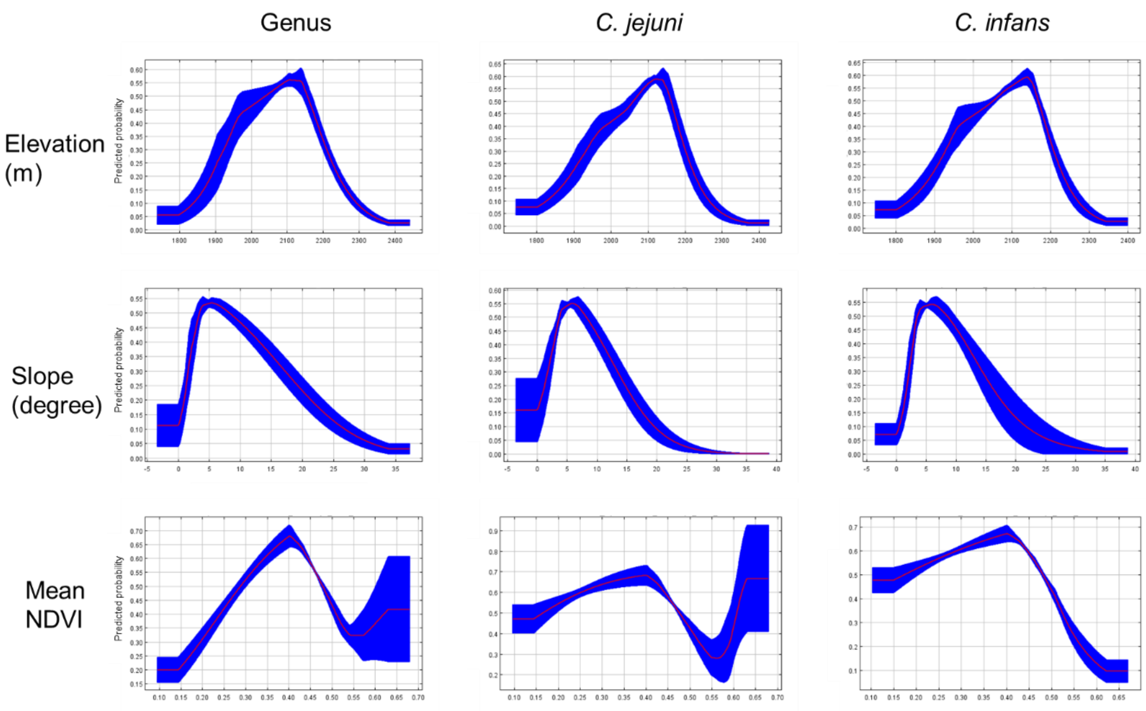

Figure S3. Response curves of three most important environmental covariates (elevation, NDVI, and slope) in the MaxEnt model. Blue ribbons represent the 95% confidence envelopes of the ten MaxEnt experiments.
